# End Tuberculosis: Challenges and Opportunities

**DOI:** 10.1155/2024/2307742

**Published:** 2024-09-04

**Authors:** Sarman Singh

**Affiliations:** ^1^ All India Institute of Medical Sciences, Bhopal, India; ^2^ Aarupadai Veedu Medical College, Pondicherry, India

Tuberculosis (TB) is one of the deadliest infectious diseases killing millions of people every year. The World Health Organization (WHO) is aiming to eliminate TB by the year 2030. However, the target looks very difficult if not impossible. To achieve this goal, a multipronged approach is required, but most importantly, clarity from WHO needs to be issued to the inventors and pharma-enterprises to invest in rapid and point-of-care serological tests. Without prompt diagnosis of infected individuals and active case finding through such RDTs, the target may look even far more distant.

TB is one of the world's oldest and deadliest infectious diseases and still continues to kill millions of people every year. WHO is committed to end TB by 2030 [1] while some countries like India have proactively advanced this target to end TB by 2025 [2]. Though ending TB in India in the next 1 year is seemingly a herculean task, nonetheless, this optimistic short duration was questioned even at the time of declaration [3]. However, various stakeholders are working on various fronts, yet the progress in achieving the targeted goal is far, especially after the COVID-19 epidemic [4]. There are several reasons why this elimination of TB is far more difficult than a few viral diseases that the world could eliminate. The vaccination could be considered the most crucial weapon in these viral diseases, beside improvement in other health indices.

An editorial in the November 23 issue of Lancet Infectious Diseases focusing on TB control through vaccine is worth reading [5]. The editorial very aptly mentions that the COVID-19 epidemic has taught us its negative as well as positive sides. On one hand, TB case detection rates were compromised due to a shift of manpower and other resources in COVID-19 management, but it has also given us hope for speedy vaccine development for various infectious diseases including TB. The editorial mentions about the targets set in the UNGA meeting on September 22, 2023 for the next 5 years including the formation of the TB Vaccine Accelerator Council. The director general of WHO also highlighted the recent WHO-commissioned study that estimates that a vaccine with 50% efficacy can avert up to 76 million new TB cases and 8·5 million deaths, in the next 25 years. However, it will require a budget of US$41·five billion. If the new vaccine is aimed to be 75% efficacious, it can avert up to 110 million new TB cases and 12·3 million deaths, but much more funding shall be required [6].

The BCG is the only currently approved and used vaccine against TB, though more than 32 vaccine candidates are in the pipeline of development. The BCG vaccine is more than 100 years old, and more than five seed lots are being used to prepare this vaccine in various countries, with theoretical differences in the efficacy of each seed lot [7]. Though in old reports, it was reported to be highly protective, but recent meta-analyses have shown its very limited role in protecting against pulmonary TB and averting TB-associated deaths. It is also shown unambiguously that its protective role vanishes after 5 years [8], and therefore, newer vaccines need to be developed, and their protective roles should be evaluated not only in infants and children but also in adults.

Vaccine development is undoubtedly a permanent long-term vision; however, no single approach is going to achieve the goal of ending TB. I strongly believe that the role of early and rapid diagnostic tests cannot be undermined. If the timely diagnosis at affordable cost is not made available at the doorstep to the affected people, especially those living in low- and middle-income countries often with high TB burden, infected patients, often presymptomatic, will continue to spread the infection, and prophylactic measures in these counties may not bring the desired fruits. Therefore, the emphasis should be given on the development and speedy evaluation of cost-effective and early diagnostics as part of all elimination strategies. A point-of-care test with remarkably high sensitivity can serve as a TB management triage and could prove a strong weapon in the anti-TB armamentarium. These tests can be developed even with slightly low specificity but can be a boon for the triage strategy. These screening tests should be rapid and point-of-care cassette or strip-based tests (Figure 1). There are several scientific groups, and even some manufacturers are willing to develop such tests [8, 9]. However, these pharmaceutical companies are holding back their enterprises in light of the WHO ban on serological tests [10]. Even though the WHO ban 12 years ago on the then-commercialised diagnostic kits was applauded, the finer lines written in the policy statement by the WHO for new research have subdued. Several research and development organizations and funding agencies are unclear about the WHO policy. We strongly believe that the search for novel antigens and the development of such point-of-care triage tests should be encouraged, and a clear message is conveyed by the WHO, to the respective national TB control organizations and funding agencies, especially in high TB burden countries, encouraging the search for new biomarkers including serological tests. It is re-emphasized that only a combined approach to screening the infected, tracking the laboratory, confirmed index cases and their contacts, and offering supervised, appropriate, and uninterrupted treatment and vaccination of the at-risk population can eliminate the TB.

*Sarman Singh*

## Figures and Tables

**Figure 1:**
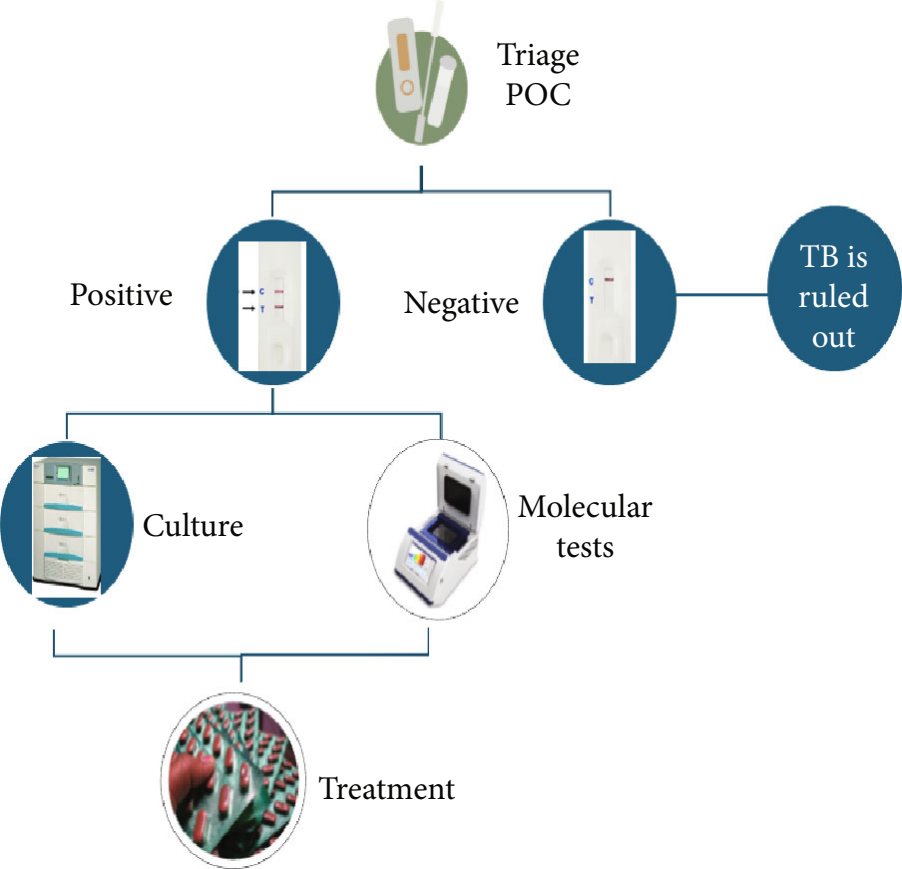
Algorithm for screening TB-suspected population using triage strategy.

## Data Availability

Data sharing is not applicable to this article as no datasets were generated or analyzed during the current study.
